# Young Children’s Understanding of Helping as Increasing Another Agent’s Utility

**DOI:** 10.1162/opmi_a_00183

**Published:** 2025-01-23

**Authors:** Laura Schlingloff-Nemecz, Barbara Pomiechowska, Denis Tatone, Barbu Revencu, Dorottya Mészégető, Gergely Csibra

**Affiliations:** Department of Cognitive Science, Central European University, Vienna, Austria; TUM School of Social Sciences and Technology, Technical University of Munich, Munich, Germany; Centre for Human Brain Health, Centre for Developmental Science, School of Psychology, University of Birmingham, Edgbaston, Birmingham, UK; School of Psychology, Faculty of Health, University of Plymouth, Plymouth, UK; Cognitive Neuroimaging Unit, CEA, INSERM, Université Paris-Saclay, NeuroSpin Center, Gif-sur-Yvette, France; Department of Psychological Sciences, Birkbeck, University of London, London, UK

**Keywords:** helping, action understanding, naïve utility calculus

## Abstract

Instrumental helping is one of the paradigmatic “prosocial” behaviors featured in developmental research on sociomoral reasoning, but not much is known about how children recognize instances of helping behaviors or understand the term ‘help’. Here, we examined whether young children represent helping as a second-order goal and take it to mean increasing the utility of another agent. In Study 1, we tested whether 12-month-old infants would expect an agent who previously helped to perform an action that reduced the Helpee’s action cost. We found that while infants expected agents to act individually efficiently (Experiment 1C), they did not expect the agent to choose the action that maximally reduced the Helpee’s cost compared to an action that reduced the cost less (Experiment 1A) or not at all (Experiment 1B). In Study 2, we examined whether three-year-old preschoolers (1) maximize a Helpee’s cost reduction when prompted to help in a first-person task, and (2) identify in a third-party context which of two agents, performing superficially similar behaviors with varying effects on the Helpee’s action options, actually helped. Contrary to our predictions, preschoolers did not help in a way that maximally reduced the Helpee’s cost in (1). In (2), however, they indicated that the agent who reduced the Helpee’s action cost was the one who helped. Taken together, these results support the proposal that, at least by preschool age, children possess a second-order utility-based concept of helping, but that they may not exhibit efficiency when choosing their own helping actions.

## INTRODUCTION

Helping is an important and ubiquitous type of human social interaction. It occurs frequently in the environment of infants and young children, both with their own involvement (as recipients or providers of help) or without (as bystanders of helping interactions). Accordingly, a large body of research suggests that very young children not only help adults with simple tasks (Warneken & Tomasello, 2006), but also reason about observed helping actions and generate social evaluations on this basis (Jara-Ettinger, Tenenbaum, & Schulz, 2015; Schlingloff-Nemecz et al., 2023; Van de Vondervoort & Hamlin, 2016).

However, not much is known about *how* children represent helping. The developmental literature has mostly focused on the types of inferences that the observation of a helping event licenses (e.g., about the cooperativeness of an agent), without addressing how children learn to identify this event in the first place. For example, it has been studied whether and when children prefer or positively evaluate a helpful character (Franchin et al., 2019; Kenward & Dahl, 2011; Kishimoto et al., 2018; Li & Tomasello, 2018; Vaish et al., 2010; Van de Vondervoort & Hamlin, 2017), what role an agent’s intentions play in such evaluations (Van de Vondervoort & Hamlin, 2018), or when children deem helping to be obligatory or desirable (Dahl et al., 2020; Eisenberg-Berg & Neal, 1981; Hepach et al., 2012, 2016; Kim et al., 2014; Sierksma et al., 2014). These studies show that children from around 3 years of age interpret helping as a positively valenced behavior whose occurrence can elicit moral reasoning, but they don’t reveal what concept of helping children draw on in these tasks.

Further, many studies probing children’s reasoning about helping interactions have used explicit linguistic primes to label cooperative interactions, and as such fall short of telling us whether children would spontaneously categorize these behaviors as helping. They often feature vignette stimuli that make explicit reference to one agent helping another (Dahl et al., 2020; de Cooke, 1992; Eisenberg-Berg & Neal, 1981; Nucci et al., 2018; Sierksma et al., 2014; Weller & Hansen Lagattuta, 2013), or visual stimuli followed by an experimenter prompt that includes the term “help” (Paulus & Moore, 2011). Even once children understand and use this term, it is unclear what they take it to refer to.

A large body of research attests to infants’ ability to draw sophisticated inferences about agents’ dispositions and relationships (Kuhlmeier et al., 2003; Rhodes et al., 2015) and express social evaluations of Helpers (e.g., Hamlin, 2013, 2014; Hamlin et al., 2007, 2011, 2013; Hamlin & Wynn, 2011; Woo et al., 2017; but see Lucca et al., 2021; Salvadori et al., 2015; Schlingloff et al., 2020) when confronted with helping scenarios. While this evidence tacitly presupposes that infants from early in their first year of life leverage a concept of helping to form dispositional evaluations of third parties, it remains silent with regards to the mechanisms underlying this concept and the specific normative expectations it foregrounds.

Here, we hypothesize that children access a concept of helping that is grounded in a general framework of action understanding via naïve utility calculus (Jara-Ettinger et al., 2016; Powell, 2022). The naïve utility calculus is based on an assumption of rationality (i.e., agents behave in a way that maximizes their utility) and a reverse-engineering process through which the internal states that caused the observable action—or the goal states that justify it—can be probabilistically inferred by an observer (Baker et al., 2009). For example, if someone approaches the front door of a house, turns the door knob, knocks on the door and waits a few minutes before walking around the house and entering through the back door, an observer can infer the agent’s goal (to go into the house) along with her mental states (a mistaken belief that the front door is open or that someone will open it for her; a true belief that the back door is unlocked) and other features of the situation (the agent doesn’t have keys). Research suggests that a naïve utility calculus supports action understanding at an early age (Csibra et al., 2003; Gergely & Csibra, 2003; Jara-Ettinger, Gweon, et al., 2015) and enables infants and children to draw rich inferences in this domain (Jara-Ettinger, Tenenbaum, & Schulz, 2015; Liu et al., 2017, 2022). Social actions, too, can be represented in terms of the costs and benefits incurred by the agents involved in them: here, the target of an action is another agent’s utility (Powell, 2022). For example, when sharing a resource with a social partner, the agent invests effort and loses access to the resource in order to provide a material benefit to the partner; when comforting a partner in distress, the agent increases the partner’s utility by improving her affective state.

In this framework, helping can be described as a type of action where one agent (the Helper) derives a reward from increasing the utility of another agent (the Helpee) by intervening on the Helpee’s action constraints (Schlingloff-Nemecz et al., 2023; Ullman et al., 2009). While in nonsocial instrumental actions, an agent manipulates an object to directly maximize her own utility (e.g., opening a door to walk through it), in helping actions an agent does so indirectly by increasing the *Helpee’s* expected utility. An action can thus be considered helping when its goal is to increase the utility of the Helpee’s action (either by lowering her cost or by increasing her rewards). Helping is therefore an action with a second-order goal, and a representation of helping is hierarchical as it embeds the utility function of the Helpee into that of the Helper. In this way, helping differs from other prosocial actions with first-order goals (e.g., the goal of giving is to donate a resource to a recipient), which may be identified and represented through different mechanisms, such as action schemas (Tatone & Csibra, 2024; Tatone et al., 2015).

Besides the utility-based account of understanding third-party helping actions, leaner proposals have been put forward in the literature, in particular to account for infants’ behavior in tasks like the one by Hamlin et al. (2007). For example, Spelke and Powell (Powell & Spelke, 2018; Spelke, 2016) have argued that infants may initially view observed helping events as instances of imitation, insofar as in most of the scenarios adopted, Helpers partly reproduce the same actions that the Helpee performed on their goal object (for similar arguments, see Benton & Lapan, 2022; Premack & Premack, 1997). However, it is unlikely that such minimal accounts can account for the sophisticated sociomoral reasoning competences of young children (and possibly infants) reviewed earlier. Moreover, these theories provide no explanation for how and when in development children might acquire a mature concept of helping, which encompasses cases in which agents take on dissimilar and complementary roles (e.g., opening a box vs. retrieving the object inside).

A few studies to date seem to suggest that preschool children and even toddlers can recruit a naïve utility calculus when interpreting helping behavior. In a study by Jara-Ettinger, Tenenbaum, and Schulz (2015), two-year-olds used the anticipated cost that an agent would incur in assisting someone with a task (activating a toy) to evaluate their prosociality: They judged a competent agent who refused to help as less nice than an incompetent one, presumably because they took the relatively higher effort that the incompetent agent would have to invest as a reasonable excuse to refrain from helping (see Sierksma et al., 2014). Jara-Ettinger et al. (2020) found that 4- to 5-year-old children expected Helpees to consider the prospective costs of a Helper: Here, children inferred that a Helpee requesting aid from two potential Helpers was likely addressing the one for whom it would be less effortful to help (i.e., the one who was physically closer). However, when that agent was unable to help, children reasoned that the Helpee was addressing the other agent (cf. Paulus & Moore, 2011).

While these studies suggest that children consider *Helpers’* action costs when engaging in social reasoning and evaluation, they do not directly examine whether children take helping to be directed at increasing the utility of the *Helpee*. Tentative evidence for this possibility comes from Bridgers et al. (2020), where 3- to 5-year-olds predicted a Helpee to express gratitude towards a Helper who prevented a relatively larger counterfactual loss for an agent. Further, in Bennett-Pierre et al. (2018), 3- to 5-year-olds directed a puppet to help an agent faced with a difficult tower-building task over another faced with a comparatively easier one. Relatedly, Woo et al. (2024) found that 16-month-old toddlers prefer a Helper who assisted one of two potential recipients facing a more costly task (i.e., pushing a boulder on a steep hill). This evidence suggests that children expect helping to be preferentially directed towards agents who face higher prospective costs of goal fulfillment.

However, these studies do not conclusively settle the question whether young children take the goal of helping to be increasing the Helpee’s utility. First, children may assume that agents facing a more difficult task are more likely to fail, and thus in greater need of assistance that increases the chance of success, but may not necessarily take the goal of helping to make task completion easier for the Helpee. Second, even if children may expect agents to direct helping towards more difficult tasks, such an expectation does not reveal what features of the action are essential to consider it an appropriate instance of helping.

To address this question, we derived several predictions from the hypothesis that infants and children would apply a hierarchical, utility-based concept of helping. (1) Children should recognize helping as an instrumental action that increases the utility of another goal-directed agent. (2) They should recognize helping also when the action reduces the costs of fulfilling a goal that the Helpee could have realized on her own. (3) They should not require perceptual cues indicating that Helper and Helpee are coordinating or affiliated. Finally, (4) they should be sensitive to degrees of helpfulness. In other words, they should expect a Helper to select an action that, at the same costs to herself, brings the highest utility increase for a Helpee.

We first conducted a series of looking-time experiments to examine whether 12-month-old infants would interpret an action decreasing a Helpee’s action costs as helping, which we report here as Study 1. In these experiments, we familiarized infants with an agent (the Helpee) approaching an object either via (a) a long path, or (b) a short path made accessible by another agent (the Helper) by pushing an obstacle blocking the Helpee’s direct access to it. The test events featured two obstacles: one which, as in familiarization, blocked the shortest path to the object, and another, which either blocked a relatively longer path (Experiment 1A) or was located elsewhere in the scene and did not obstruct the Helpee’s path (Experiment 1B). We hypothesized that infants would expect the Helper to move aside an obstacle that blocked the Helpee’s shortest path to the goal. Moreover, we conducted a control experiment (Experiment 1C) to assess whether infants, after being familiarized with the same stimuli as in Experiment 1B, would expect the Helpee to act individually efficiently.

The results of the infant experiments subsequently prompted us to investigate whether older children would understand helping as directed towards another goal-directed agent’s utility. We examined this question in two contexts: (1) one in which children themselves were asked to help someone, and (2) another where they observed an agent’s helping behavior and had to infer its goal. In context (1), children were presented with an agent whose goal was to collect tokens of a target object. At test, the agent was prevented from reaching her goal by obstacles blocking the way. Children could help by moving aside one of two obstacles, either an obstacle that blocked a short path to the object or an obstacle that blocked a long path to the object, respectively. In context (2), children saw the same agent with two other agents: One removed an obstacle blocking the shorter path to the object, whereas the other removed an obstacle that was not blocking any path or pushed an obstacle in front of the short path, thus forcing the Helpee to take the longer one. Children were then asked to indicate who helped. In both tasks, we considered their response ‘correct’ if it designated an action that decreased the Helpee’s expected costs relative to the alternative option.

## STUDY 1

In the experiments of Study 1, we tested whether 12-month-old infants represent helping as a second-order goal directed at increasing the utility of another agent. We targeted this age group, as there is previous evidence suggesting that 12-month-olds may already possess prerequisite capacities for a second-order, utility-based concept of helping. First, they have a robust understanding that agents should act efficiently by minimizing their action costs (Gergely & Csibra, 2003). Second, they have been found to preferentially reach for Helpers over non-Helpers, and this preference seems grounded in a sophisticated and nuanced interpretation of Helpers’ motives (e.g., Hamlin, 2013; Hamlin et al., 2007; see Schlingloff-Nemecz et al., 2023 for a review).

We include here only a brief summary of the experiments, as their results are somewhat inconclusive and led us to design Study 2 as a follow-up with older children. Details about the participants, procedure, stimuli, and results can be found in the Supplementary Materials. The stimuli and data are accessible at https://osf.io/adyqn/.

### Experiment 1A

**Figure 1.**
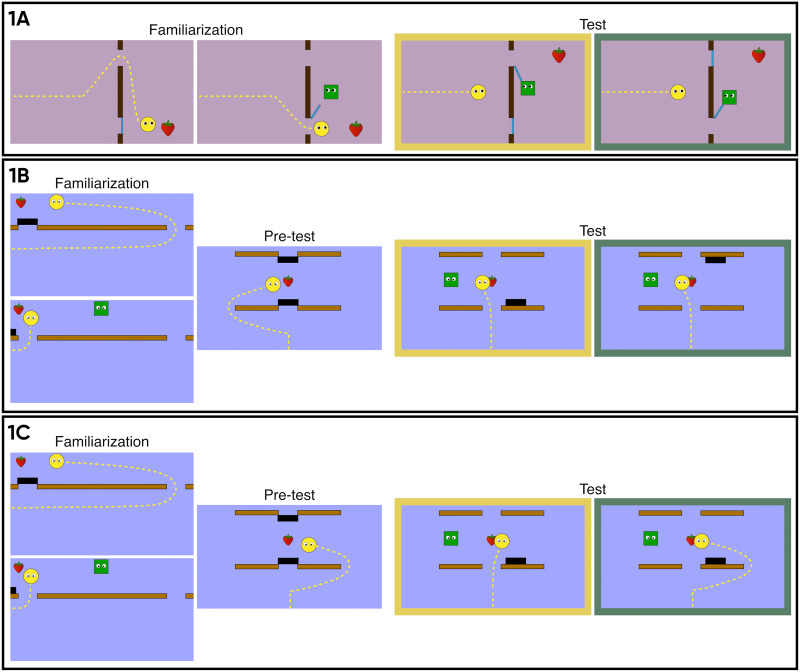
Screenshots from the experimental stimuli of Study 1. Dotted arrows indicate the agents’ motion paths. In the familiarization trials (left), the Helpee agent (yellow circle) approached the strawberry on a long path when he was alone and took a shorter path when the Helper (green square) was present, as the latter removed an obstacle covering a gap in the wall. Depicted are examples of the familiarization trials that infants watched; in the experiment, the layouts of the scenes and corresponding movement paths varied across familiarization trials. In the pre-test trials (Experiments 1B and 1C), infants saw the Helpee approach the goal in a layout that was nearly identical to that shown at test. The test trial events (right) differed by experiment; the left image on the yellow background depicts the Consistent test trial, the right image on the green background the Inconsistent test trial. In Experiment 1A, the Helper either opened the door that allowed the Helpee to take the shorter path (Consistent) or the door that freed the longer path (Inconsistent). In Experiment 1B, the Helper either removed an obstacle that blocked the Helpee’s direct path (Consistent) or an obstacle that was located elsewhere in the scene (Inconsistent). In Experiment 1C, the Helper helped in both test trials, but the Helpee either efficiently approached the goal on a direct path (Consistent) or detoured around the side of the wall (Inconsistent).

In Experiment 1A, infants watched an agent (the Helpee) approach a goal object by detouring around a barrier. During familiarization, the Helper (whenever present) reduced the Helpee’s action cost by opening a door and thus allowing him to take a shorter path. At test, two doors were blocking direct access to the goal, one closer and one further away from the goal, but equidistant from the Helper (Figure 1, top row). The Helper either opened the closer door, which freed a relatively shorter path, as during familiarization (“Consistent” test trial), or opened the further-away door, freeing a longer path to the object (“Inconsistent” test trial). If infants interpreted the Helper’s goal during familiarization as directed at maximizing the Helpee’s utility, they should find it inconsistent with this goal when the Helper, at the same cost to himself, freed the longer path to the goal object.

**Figure 2.**
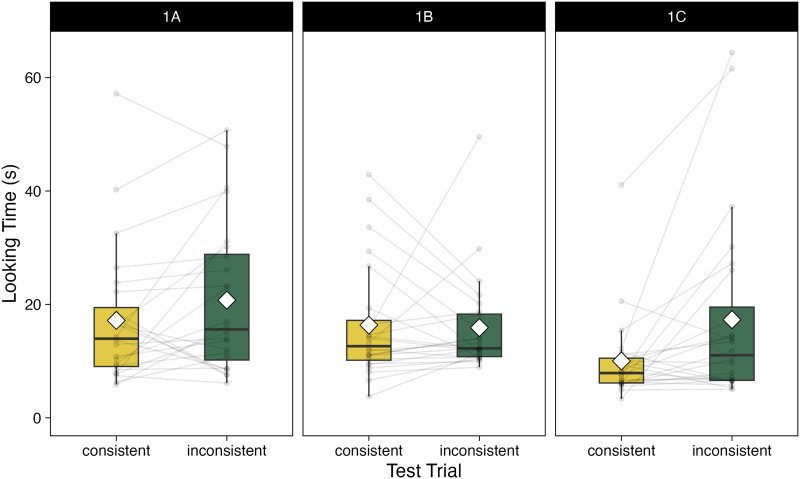
Results from Experiments 1A-C, Study 1. Box plots of average looking times (in seconds) to the test events. Light grey lines connect the looking times of individual participants, white diamonds indicate means, horizontal lines indicate medians, boxes indicate middle quartiles, and whiskers indicate data points within 1.5 times the interquartile range from the upper and lower edges of the middle quartiles.

We tested 24 12-month-old infants in a violation-of-expectation looking-time paradigm with 8 familiarization trials and 2 test trials. Looking times recorded during the test trials were base-10 log transformed, and we calculated Bayes Factors following the method recommended by Csibra et al. (2016) in this and the following experiments from Study 1. We obtained a Bayes Factor of 0.54, which constitutes anecdotal evidence for the null hypothesis of no effect (*M*_consistent_ = 17.78 s, *SD*_consistent_ = 11.97 s; *M*_inconsistent_ = 20.75 s, *SD*_inconsistent_ = 13.38 s; Figure 2).

### Experiment 1B

In Experiment 1A, infants had to compare the effects of two actions that both ultimately allowed the Helpee to reach her goal, although one of them at a relatively lower cost for the Helpee (thus, maximizing his overall utility). We did not find that infants expected the Helper to act in a utility-maximizing manner towards the Helpee. One possible interpretation of this result is that, by allowing the Helpee to reach the object, both actions could be considered well-formed instances of helping, in spite of the different costs the Helpee has to incur to fulfill his goal. If infants do not compare action options but simply assess whether an action reduces the Helpee’s action cost, they should be able to appropriately identify helping when a scenario in which the Helpee does not receive any assistance is directly compared with one in which the Helper intervenes to reduce his costs.

Experiment 1B was designed to examine this possibility. Here, we tested whether 12-month-old infants would expect a Helper to perform an action that reduced the costs of the Helpee’s goal fulfillment compared to a superficially similar-looking action that left these costs unaltered.

Infants were again familiarized with a scenario where a Helpee had to take a longer path to reach a reward when he was by himself, but could take a shorter path thanks to the Helper’s assistance. At test, the Helper removed an obstacle, whose location differed across test trials: In the Consistent test event, the obstacle was obstructing the Helpee’s direct path to the goal object, and thus its removal reduced the Helpee’s cost, whereas in the Inconsistent test event, the obstacle was not blocking the Helpee’s path (Figure 1, middle row), making its displacement irrelevant to the Helpee’s goal.

We tested 24 12-month-old infants in a violation-of-expectation looking-time design with 8 familiarization trials, 1 pre-test and 2 test trials. We obtained a Bayes Factor of 0.11, which constitutes substantial evidence for the null hypothesis (*M*_consistent_ = 16.35 s, *SD*_consistent_ = 10.28 s; *M*_inconsistent_ = 15.92 s, *SD*_inconsistent_ = 8.84 s, Figure 2).

### Experiment 1C

The results from Experiment 1B did not support the hypothesis that infants possess a concept of helping as directed at increasing the Helpee’s utility. To rule out the possibility that the stimuli used were generally unsuitable for eliciting goal attribution to the Helpee in 12-month-olds (which is a precondition for interpreting the Helper’s action as helping), we conducted a control experiment, in which we tested whether infants successfully attributed to the Helpee the goal of approaching his object. Participants of the same age group first watched the familiarization stimuli from Experiment 1B, and subsequently saw two test events where the Helpee either moved towards the goal object directly (Consistent test event), or by taking an unnecessary detour (Inconsistent test event; Figure 1, bottom row). The Helper behaved identically across test trials, in both cases removing an obstacle which blocked the Helpee’s direct path. We predicted that if infants ascribed an instrumental goal to the Helpee, they should look longer when he behaved inefficiently.

We tested 24 12-month-old infants in an experiment that was identical to Experiment 1B, save for the two test trials. We obtained a Bayes Factor of 323.59, which constitutes strong evidence in favor of the hypothesis; thus, infants looked longer at the Inconsistent test event depicting an inefficient action compared to the Consistent test event (*M*_inconsistent_ = 17.32 s, *SD*_inconsistent_ = 16.54 s, *M*_consistent_ = 10.02 s, *SD*_consistent_ = 7.59 s; Figure 2).

### Study 1: Discussion

The aim of the experiments reported here was to investigate whether infants understand helping as an action whose goal is to lower or minimize another agent’s action costs. The results, taken together, do not support this hypothesis. In fact, the Bayes Factors for Experiments 1A and 1B provided some evidence in favor of the null hypothesis of no effect: infants did not distinguish between an event in which a Helper maximized or increased a Helpee’s utility, and one in which the Helper performed a similar-looking action which did not impact the Helpee’s goal or did so suboptimally. Experiment 1C demonstrated that infants familiarized to the same stimuli as those in Experiment 1B successfully ascribed an instrumental goal to the Helpee in this scenario, ruling out the possibility that the stimuli or experimental procedure failed to elicit any kind of goal attribution.

While this pattern of results can be interpreted in various ways (see Supplementary Material for a detailed discussion), we tentatively conclude that infants struggle to deploy a concept of helping as a second-order goal directed at the increase of another agent’s utility.

Following up on these findings, we designed a study with older children, featuring animated stimuli similar to those used in Study 1, where verbal prompts were employed. The aim was to probe whether preschoolers’ understanding of the term “help” would map onto a second-order concept of helping.

## STUDY 2

### Methods

The procedure and analysis plan were preregistered (https://osf.io/v4yeg). All materials, including the stimuli and testing script, data, and model code, can be accessed at https://osf.io/ts84j/.

#### Participants.

Our sample consisted of 64 Hungarian-speaking children (age range: 3;0 to 4;0, mean age: 42.8 months). An additional 16 children participated in the experiment but were excluded for showing a side bias by indicating the same side (left or right) across all four trials (*n* = 10), failing to provide a valid response in at least one trial per block (*n* = 5), or technical failure (*n* = 1). Participants were recruited through the lab’s database. Informed consent was obtained from caregivers before the testing procedure, and children were asked if they wanted to participate. The study received full ethical approval from the university’s ethics committee.

#### Apparatus.

The experiment was conducted remotely via video chat (Zoom), with stimuli presented on the participants’ web browser via Slides.com. The child saw the stimuli videos in near full-screen size, with a small window above the stimuli in the top center of the screen showing the experimenter. Caregivers were asked to hide the window showing themselves and the child, to minimize distractions. The Zoom call was recorded and the recording, which included the video of the child, was saved to the experimenter’s computer for later off-line coding of responses.

Video stimuli were created using the Blender open-source 3D computer graphics software toolset, version 2.92.0 (https://www.blender.org/). We used an audio cue from the publicly available stimuli of Liu et al. (2017).

#### Procedure and Stimuli.

The experiment consisted of 3 phases: a warm-up task and two experimental blocks.

##### Warm-Up Task.

In the warm-up task, children were shown images of animals with colors matching the ones in the experimental stimuli, and were asked to identify them by pointing (e.g., the experimenter asked, “Can you show me where the horse is?”) as well as to label their color. Children were then introduced to an agent called “kobo” (a green circle with eyes), who likes apples and is sometimes forced to detour rocks to collect them.

##### Experimental Blocks.

Each experimental block contained a familiarization followed by two test trials.

##### Block 1.

**Figure 3.**
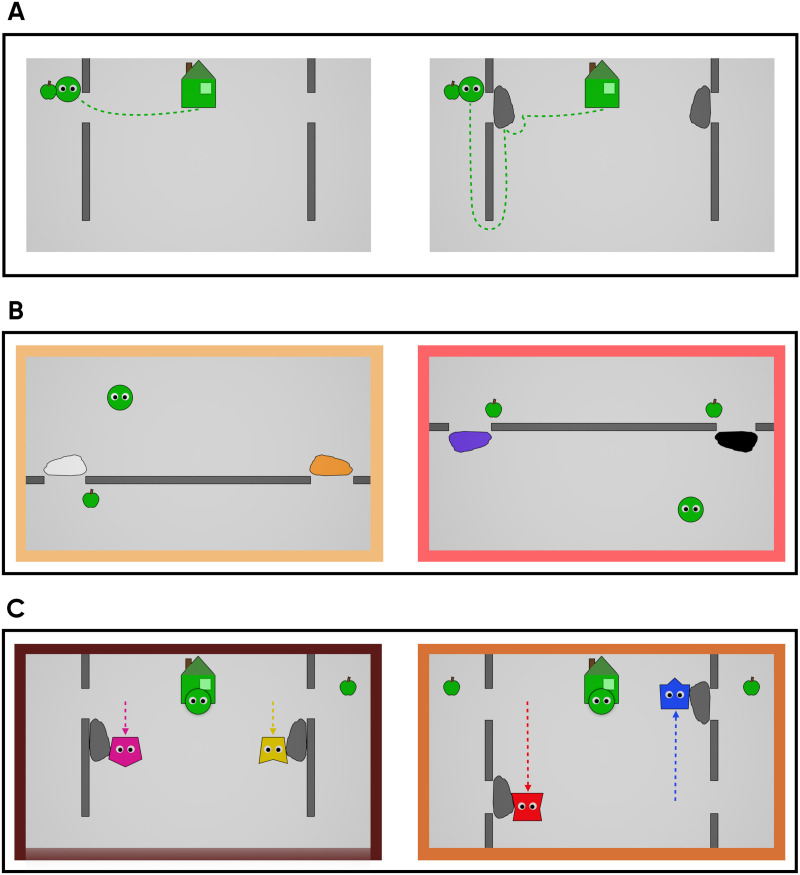
Screenshots from the experimental stimuli; dotted arrows indicate the agents’ motion paths. In the Familiarization video (A), the protagonist agent (green circle) either approached the goal on the most direct path through a gap in the wall (left), or, when a rock obstructed this path, first attempted to move the obstacle, and then detoured around the bottom of the wall (right). In the Block 1 test events (B), children were asked to help by moving aside one of two rocks, blocking the paths to a single goal (left) or two possible goals (right). In the Block 2 test events (C), an agent who helped by making a shorter path to the goal accessible (left: yellow agent, right: red agent) was contrasted with one who performed an action that either did not affect the green agent’s action plan (left: pink agent; “help vs. irrelevant” trial), or one that hindered it by blocking the shortest path (right: blue agent; “help vs. hinder” trial).

In the first experimental block we probed whether children would help an agent in such a way that the agent’s action cost is minimized. At the beginning of the block, a familiarization video was played (Figure 3A). This video contained the house of the kobo in the top center, and vertical walls on the left and right side of the scene. Each wall was interrupted by a short gap approximately at the house level. The walls ran from the top of the display nearly all the way to the bottom, leaving a small space for the agent to detour them. In the first part of the familiarization video, the kobo exited his house, and an apple appeared in the scene behind the gap in one of the walls. The kobo looked at the apple, then approached it through the gap, made contact with it, and carried it back to the house. The second part of the familiarization video started the same way, but just before the kobo reached the gap in the wall to approach the apple, a gray rock appeared in front of the gap. The kobo bumped against the rock twice as if attempting to move it, then detoured around the unobstructed, long end of the wall at the bottom of the screen, thus taking a longer path than before. He again took the apple back into his house along the same longer path.

After this familiarization, children were presented with two test trials (Figure 3B). In each of the trials, children saw the kobo in a new layout with a single horizontal wall placed directly below it. The wall was interrupted by two gaps, one of them closer to the location of the kobo. In one of the test trials (“one goal”), a single apple appears behind the closer gap. The kobo looked at it, but before he started moving to approach it, two rocks of different colors (orange and white) appeared in front of the gaps. At this point the experimenter told the child that they could help the kobo by moving aside one of the rocks, and asked which one should be moved. After the child responded, the experimenter played a video clip where the rock indicated by the child moved aside, and the kobo went to the apple. In the other test trial (“two goals”), two apples appeared, one behind each of the gaps respectively. The apple located further away always appeared first. Again, after the kobo looked at both of them in turn as they appeared, two rocks (in different colors: black and purple) appeared in front of the gaps. The colors of these rocks differed from those in the previous trial to avoid carryover effects. The child was again asked to help by moving one of the rocks, and after she responded, the experimenter played the corresponding video clip where the rock moved aside and the kobo approached the apple. Note that in this task, either option enabled goal completion: regardless of the distance from the kobo, the displacement of either obstacle made a previously inaccessible apple reachable. In this sense, the structure of the tasks in Block 1 was similar to those of Experiment 1A with infants: in both cases, helping consisted in allowing a Helpee to reach a previously inaccessible goal, either by freeing a shorter or a longer path to the object.

##### Block 2.

The second experimental block also contained two test trials (Figure 3C). Here we tested whether children could correctly identify which of two agents helped, that is, who acted in a way that reduced the Helpee’s action cost. The test trials in this block were preceded by the same familiarization video that was used in Block 1. In the first test trial (“help vs. irrelevant”), the video’s layout was identical to the one in the preceding familiarization clip. Here, two (grey) rocks were already present at the onset of the video, each blocking a gap in the wall. In this video, after the green kobo exited his house, two other kobos (pink and yellow) entered from the bottom of the screen. An apple appeared on one side of the screen, and all kobos looked at it (first the green kobo, then the other two simultaneously). After this, the pink and yellow kobo each moved towards one of the rocks and pulled it downward freeing the two gaps, at which point the video ended. The video was repeated a second time, and the experimenter then asked the child which kobo helped. In this trial, while one kobo reduced the costs of the Helpee (relative to the costs he would incur to approach the apple if unaided), the other agent didn’t, since the removal of the other rock was irrelevant to approaching the apple.

The second test trial (“help vs. hinder”) had a similar structure, but the layout of the scene was slightly different. The two vertical walls extended to the top and bottom boundaries of the screen but were interrupted by two gaps, respectively. Here, the wall could only be crossed by passing through one of the gaps; there was no way to detour it. On one side, a rock covered the bottom gap, on the other side, a rock covered the top gap. In this video, two apples appeared, one behind each of the top gaps in the walls. When the novel kobos (blue and red) moved the rocks aside, one of them moved the rock covering the top gap so that it now covered the bottom gap, while the other moved the rock from the bottom to the top gap. This video was also repeated a second time and the child was then asked by the experimenter which kobo helped. In this case, while one kobo made the shorter path to the apple accessible, reducing the target’s agent costs (relative to the ones he would have incurred to reach the apple if unassisted), the other obstructed it. In sum, one kobo helped the target agent, while the other hindered.

Note that in Block 2 (in contrast to Block 1), children did not see the continuation of the video after the rocks were moved, and thus did not see the consequence of the kobos’ actions on the behavior of the green kobo (i.e., which path he took). The rationale for this was that if children saw the kobo complete his action before responding, the direction of the kobo’s movement (i.e., towards the left or right side) and the physical proximity of agents could have biased their responses, by inducing them to select the kobo approached by the green kobo to as the one who helped. We also did not show the outcomes after the children responded, to avoid them interpreting the kobo’s (in)congruent behavior as (dis)confirmatory feedback.

The structure of the test trials in Block 2 resembled those of Experiment 1B with infants: while the Helpee could in principle reach the goal by himself, the Helper’s action allowed him to do so at lower costs (by crossing a shorter path).

The key events in all videos were accompanied by different sound cues.

We counterbalanced the following factors in the stimuli: (1) the colors of the rocks in Block 1 and of the agents in Block 2; (2) the order of trials in Block 1 (“one goal” first vs. “two goals” first); (3) the location of the apple in the familiarization video (left vs. right); (4) the location of the correct response, relative to the location of the apple in the preceding familiarization video, in the first and second test trial in a block (same-same, same-different, different-same, different-different). This resulted in 32 sets of stimuli.

We decided not to counterbalance the order of blocks, and the order of trials in Block 2, following conclusions drawn from pilot data. We piloted different versions of the experiment (with a total of *n* = 46 children participating in the piloting phase), varying, among other things, the order of trials. During piloting, (1) children gave more correct responses in the “who helped” trials when these appeared in the second block, but the accuracy in the “how to help” trials did not differ depending on which block they were presented in; (2) children were least accurate in the “help vs. hinder” trials; and (3) the order of the “how to help” trials seemed to matter, such that children improved from the first to the second trial.

If in any of the four trials a child did not respond immediately, responded with “both”, or indicated the green kobo, the experimenter repeated the question and encouraged the child to “pick one”.

We excluded from the analysis trials (a) on which a child did not provide a codable response (e.g., by responding “none”, not responding, responding with “both”, or referring to the protagonist, even after being prompted repeatedly to identify one option), (b) where a child’s verbal response with a color label did not unambiguously pick out one of the two options, and (c) where their color labels from the warm-up task did not help disambiguate their response, and the child did not subsequently provide a response by pointing. Finally, we excluded all participants who didn’t provide a codable response in at least two trials, one from each block, and those who displayed a side bias by indicating the same side (left or right) across all four trials.

#### Analysis.

Our main question was whether children would above chance (1) choose to move an obstacle which lets the protagonist take a relatively shorter path to the goal (Block 1 test trials), and (2) respond that another agent helped who removed an obstacle which would let the protagonist take a shorter path to the goal (Block 2 test trials). The dependent variable was thus children’s choice of the “help” option in each of the four trials (coded as the “correct” response).

We analyzed the data using a Bayesian logistic regression. As predictors, we included the trial (1–4) and subject as random slopes. Each data point was modeled as a function of the participant who produced it and the trial on which it was produced. This allowed us to estimate (1) whether children responded more accurately on some trials than others, and (2) whether there was an effect of block (Trials 1–2 versus 3–4). Because we modeled trial and subject as random slopes, we could also test for a trial by order interaction for Block 1, to find out whether the order in which the trials in Block 1 were presented matters. As the aim of this analysis is parameter estimation (and not hypothesis testing), we characterize the entire posterior probability distribution for each of these estimates.

To estimate the probability of answering correctly as a function of trial type (where 0 is incorrect, 1 is correct, and 0.5 is the chance level), we computed the mean of the trial predictors as well as the 89% credible intervals (CI) around it. These CIs specify that the true parameter value lies in this interval with 89% probability. For the remaining questions, we used the posterior distributions of the predictors (trial and subject) to compute the implied probabilities of responding correctly for each child and trial. This allowed us to characterize the uncertainty around each individual data point as a function of the participant who produced it and the trial type on which it was produced.

We conducted an exploratory analysis to determine whether any of the counterbalanced factors affected children’s responses. A further exploratory analysis assessed whether children’s rate of correct responses in Block 1 predicted their responses in Block 2.

The analyses were performed in R (version 4.2.2, R Core Team, 2023), using the rethinking package (version 4.2, McElreath, 2020). Figures were created using the Wes Anderson Palettes R package (https://github.com/karthik/wesanderson).

### Results

#### Hypothesis-Driven Analyses.

**Figure 4.**
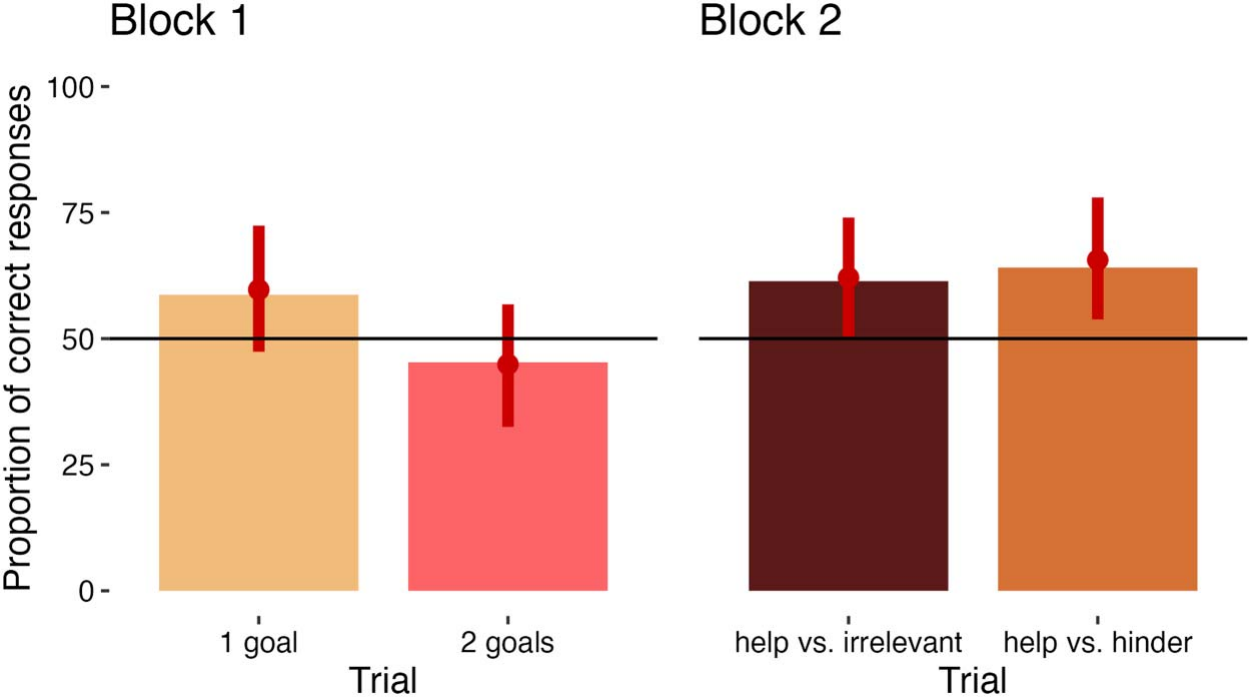
Bar plots representing the proportion of correct responses. Red error bars indicate the 89% CI of the parameter estimates, with dots indicating the means. The bars cross the chance level in both trials of Block 1, and don’t cross chance in both trials of Block 2.

In Block 1, 58.7% of responses in the “one goal” trial were correct, as were 45.3% of responses in the “two goals” trial. In Block 2, 61.4 % of responses in the “help vs. irrelevant” trial and 64.1% of responses in the “help vs. hinder” trial were correct (Figure 4).

In Block 1, the parameter estimates did not exclude chance, although for the “one goal” trial, the majority of estimates were above the chance value (“one goal” trial: mean = .598, 89% CI: [.474, .726]; “two goals” trial: mean = .449, 89% CI: [.333, .568]). In Block 2, the parameter estimates excluded chance, although for the “help vs. irrelevant” trial, this was just barely the case (“help vs. irrelevant” trial: mean = .625, 89% CI: [.51, .745]; “help vs. hinder” trial: mean = .655, 89% CI: [.539, .777]).

We also found that children were more accurate in Block 2 than Block 1. The posterior distribution of the probability to give a correct response in one block was subtracted from that in the other block, and the mean of the differences was calculated. The parameter estimate for the difference between responses in the two blocks thus generated excluded the chance level of 0 (mean = 0.101, 89% CI: [0.013, 0.187]).

#### Further Results.

We assessed whether there was an order effect in Block 1, where the order of trials was counterbalanced (2 goals first vs. 1 goal first), by checking for an interaction between trial type and order in this block. We computed two differences between the trial types (one for each order), then took the difference between these differences to check whether it is different from 0, which would signal an interaction. The 89% CI of this value included 0 (mean = .035, 89% CI: [−.062, .162]). Children’s accuracy here was thus not affected by whether a trial came first or second in the sequence.

We also checked whether children’s responses in Block 1 predicted those in Block 2, to see whether children who were more likely to be correct in one would also be better in the other. To do so, we obtained correlations between the posterior probabilities of answering correctly across the two blocks. If there was a relation between children’s responses in Block 2 and children’s responses in Block 1, the model would be able to predict Block 2 from Block 1. However, while there was a small correlation between the two blocks, the uncertainty intervals did not exclude the chance level (mean *r* value = .171, 89% CI: [−.496, .721]).

For each block, we assessed how many of those children who contributed two datapoints gave correct responses in both, neither, or only one of the trials. In Block 1, 25.4% of children were correct in both trials, 20.6% in neither trial, 33.3% were correct in the “one goal” but not the “two goals” trial, and 20.6% in the “two goals” but not the “one goal” trial. In Block 2, 38.6% were correct in both trials, 15.8% in neither, 22.8% were correct in the “help vs. irrelevant” but not in the “help vs. hinder” trial, and 22.8% in the “help vs. hinder” but not the “help vs. irrelevant” trials.

We recorded whether children in Block 2, as their first response to the question which agent helped, replied “both”. Because it is in principle possible for more than one agent to help in a given situation, we wanted to see whether children entertained such an interpretation, and whether they did so more in one of the trial comparisons than the other. In the “help vs. irrelevant” trial, 33 of 64 from the initial responses were “both”, whereas in the “help vs. hinder” trial this ratio decreased to 4 of 64.

Finally, we looked into whether side congruency between familiarization and test (i.e., the location of the apple in a familiarization trial being the same as the side of the correct response in the subsequent test trial) biased children’s responses. Children were somewhat more correct in side-congruent trials (.66) than in side-incongruent trials (.5), though this was not significant in a chi-squared test (*χ*^2^(1) = 2.578, *p* = .108). This effect was more pronounced in Block 1 (accuracy in congruent trials: .66, in incongruent trials: .45) than in Block 2 (accuracy in congruent trials: .67, in incongruent trials: .56).^1^

### Discussion

The aim of the present study was to investigate how young children interpret the term “help”, and whether they take into account agents’ costs and benefits when ascribing this goal to others. To do so, we assessed whether preschoolers (1) would help in a way that minimized the Helpee’s costs, and (2) would identify an agent who increased the Helpee’s utility by reducing his costs of goal fulfillment as the one who helped. We found that children succeeded in task (2) but not in task (1): When asked to help another agent, 3-year-olds in our study did not consistently select an action that minimized the Helpee’s costs, irrespective of whether it was directed at one or two possible goal objects. When asked to identify who helped, on the other hand, they reliably selected the agent who increased the Helpee’s utility when this agent was contrasted with an agent who hindered the Helpee; when the Helper was contrasted with an agent performing an irrelevant action, children’s responses were just above chance.

This pattern of responses is consistent with the proposal that children conceive helping as an action aimed at increasing the utility of a Helpee relative to the utility she would derive from attempting to fulfill her goal unaided. In Block 1, either intervention was helpful in this sense. Since the agent could not have reached the goal object without either obstacle being removed, even pushing away the obstacle that freed the relatively longer path resulted in a relative increase in the Helpee’s utility. This is in contrast to Block 2, where only the Helper’s action, but not that of the other agents, lowered the Helpee’s action cost by allowing him to take a shorter path to the goal object; thus, only one of the agents helped.

We found that children responded most accurately when helping was contrasted with hindering. This suggests that contrasting helping with an action yielding opposite effects on the patient’s utility may have aided children in identifying the appropriate referent concept, perhaps because it allowed them to reason by exclusion. The lack of contrastive evidence in the “help vs. irrelevant” trial may also explain why many children initially responded “both” when asked who helped in this trial. In the absence of evidence that an agent performed a harmful action, children may have defaulted on applying the term “help” also to the unrelated action, and then corrected themselves when prompted by the experimenter to identify a single referent (note however that the better performance and the lower likelihood of responding “both” in the “help vs. hinder” contrast could also be due to this trial being always presented second).

Compatible with this reading, most studies on infants’ and young children’s understanding and evaluating of helping makes use of this type of “help vs. hinder” contrastive information. Even though 6- and 10-month-olds have been found to prefer a helpful over a neutral character (Chae & Song, 2018; Hamlin et al., 2007), thus attesting to their ability to discriminate positive from null utility effects, other research on negativity bias suggests that infants may have a less ambiguous understanding of actions with negative consequences (Chae & Song, 2018; Hamlin et al., 2010). In a recent study, Wong et al. (2023) showed that contrastive information about the social effects of actions, even when presented in a counterfactual format (i.e., children generated selfish alternatives for how an agent who previously acted prosocially could have behaved), strengthened social evaluation.

We had predicted that children would help by selecting the intervention that would reduce the Helpee’s action cost more than the other one in Block 1. This hypothesis was derived from the expectation that, given identical action costs to children, they should have helped in a way that maximized their own reward (i.e., by choosing the means that maximized the Helpee’s utility). There are various reasons for why children failed to do so in our task.

One possibility is that children struggled with setting up comparisons between different representations of expected costs. Some researchers have argued that the capacity to consider multiple possibilities at the same time emerges only around 4 years of age (e.g., Leahy & Carey, 2020), so the 3-year-olds in our experiment may have failed to do so. The task in Block 2 did not require comparing mutually exclusive potential alternatives, just actions that had already been performed by different agents. It should be noted here that 3-year-olds’ failure in Block 1 echoes a recent finding by Gönül and Paulus (2021), where children of the same age did not predict that an agent would approach a goal following an efficient path. If preschoolers indeed lack the ability to compare possible alternatives of different costs, this conclusion remains to be reconciled with the large body of literature demonstrating robust expectations of efficiency in infancy (e.g., Csibra et al., 1999, 2003; Liu & Spelke, 2017; Phillips & Wellman, 2005; Scott & Baillargeon, 2013; Sodian et al., 2004). The tasks used with infants on the one hand, and with preschoolers in the present study and by Gönül and Paulus on the other, may not recruit the same cognitive processes.

Another possibility is that, even if children were able to compare alternatives, they may have appealed to a concept of helping which would have been fulfilled by either intervention. Such a concept entails that the goal of a Helper is to increase the utility of an Helpee relatively to the utility she would generate if attempting to realize her goal unaided—a criterion which both options in Block 1 satisfied. In this case, children may simply have selected one of the options at random, and since it matched their idea of helping, they did not search for other means to help that could potentially yield an even higher utility increase for the Helpee.

This account echoes, albeit loosely, the phenomenon of “ineffective altruism”, according to which people tend to be insensitive about the impact and effectiveness of their prosocial behavior (for a review: Caviola et al., 2021). In this sense, the children’s behavior may be similarly deemed “ineffective” insofar as it appeared to be satisfied by any amount of positive change (cost reduction) to the recipient. However, surface similarities aside, the lax criterion of helping that children’s indiscriminate intervention may have revealed in Block 1 is unlikely to be caused by the motivational (i.e., prioritizing interventions with reputational consequences) and epistemic (i.e., believing in the ineffectiveness of organizational over direct help) factors commonly adduced to explain ineffective altruism in adults (Caviola et al., 2020; Johnson, 2018).

A further possibility is that having children participate in a first-person helping task may not elicit their normative assessment of an action efficiency to the same extent as having them evaluate it in a third-party context. In other words, children may understand that, given equal costs to the Helper, the Helper should choose the action that maximizes the Helpee’s utility, but fail to implement this understanding in their own behavior. Other contexts, in which children are asked how helping should be done (cf. Bennett-Pierre et al., 2018), may be more suitable for eliciting corresponding responses, and we cannot rule out that children would have answered as predicted if we had phrased or framed the task differently. Previous studies have often found a “knowledge-behavior gap”, such that children’s understanding and evaluation of sociomoral actions do not always align with their own behavior (e.g., Blake et al., 2014; Smith et al., 2013). However, it should be noted that altruistic behavior in tasks eliciting a knowledge-behavior gap generally involves a personal cost for the child, which creates conflicting motivations (i.e., self-interest vs. norm-adherence), while in our task both helping actions were equally “costly” for the child (cf. Radovanovic et al., 2025).

Relatedly, children were asked to assist an animated agent in a simple grid-world, where the mechanism by which they “moved” the obstacle aside remained opaque. In a more realistic context, helping a partner with a familiar task, impeded by known constraints and costs, children may have behaved differently (cf. Bridgers et al., 2017; Buttelmann et al., 2009; Hobbs & Spelke, 2015; Paulus, 2020). Nevertheless, the aim of the present study was to identify the minimal features that an action needs to display in in order to be interpreted as helping, and, in such respect, abstract stimuli are particularly useful at assessing the sufficiency of perceptual inputs in a highly controlled manner. Moreover, such stimuli have been repeatedly used to induce a variety of representations of social goals in infants and children (Kominsky et al., 2022)

Furthermore, because children in our task were not themselves represented by a character in the grid-world, we do not know where they imagined themselves located spatially. Had they anchored themselves not at the center of the screen, but on the farthest side from the agent, they may have attempted to minimize *their own* action cost, rather than solely focusing on the Helpee’s cost.

Finally, it cannot be ruled out that there was an effect of trial order on the pattern of results we obtained: Block 2, in which children were asked to point out who helped, always came second, and the “help vs. hinder” trial, where children responded most accurately, was always presented last. It is possible that children could have learned something over the course of the experiment, for example by observing the consequences of their own helping actions on the agent’s action cost in Block 1. It should be noted, however, that they never received explicit feedback on their responses, and that there was no effect of trial order in Block 1, where this was counterbalanced.

Overall, the present results are consistent with the proposal that, around 3 years of age, children leverage a hierarchical, utility-based concept of helping. In the introduction to this paper, we laid out four predictions that follow if children possess such a concept: they should (1) recognize it as an action that increases another agent’s utility; (2) do so even when that other agent could have reached the goal alone; (3) not require specific perceptual cues to identify instances of helping; and (4) be sensitive to degrees of helpfulness. In two experiments with infants (Study 1, Experiments 1A and B), these predictions were not confirmed. In Study 2, results were mixed. Findings from Block 2 showed that children correctly identified an agent who increased the Helpee’s utility as the one who helped (1), even though the Helpee could have reached the goal unaided (at a greater personal cost) (2) and in spite of the absence of interactive cues in the stimuli (3). However, in Block 1, children did not intervene in the most helpful way possible (4). As discussed before, this may reflect that they consider any action helping which increases another’s utility, but do not distinguish the relative effectiveness of different helpful interventions, or it may be due to specific tasks characteristics earlier reviewed.

A recent paper outlined two simpler alternative concepts that young children may initially rely on (Schlingloff-Nemecz et al., 2023). One possibility is that they take the goal of helping to be making the Helpee’s goal completion possible, which we call helping as ‘enabling’. Under such a concept, helping has a qualitative effect for the Helpee but the magnitude of utility provided to the Helpee is not tracked, as long as she succeeds in the end. Supporting this conjecture is the fact that most studies with infants and children featuring helping show or describe a situation in which the Helpee fails in her goal pursuit on her own, and is able to succeed only upon receiving help (e.g., Hamlin & Wynn, 2011; Hamlin et al., 2007; Köster et al., 2016; Kuhlmeier et al., 2003). In this case, a need for help would not mean facing a relatively high cost to reach one’s goal, but a high probability that without assistance, one would fail to do so at all.

If children think that helping means making goal completion possible, they would *not* consider an action helping that increases someone’s utility if the agent could also have reached her goal alone. In Block 2 of the current study, children responded correctly above chance that an agent who reduced a Helpee’s action cost was the one who helped, even though the Helpee could have reached the goal on his own (as children saw during the familiarization video, where the Helpee detoured around the lower edge of the wall in the same layout). This finding thus provides evidence against the enabling account.

A second alternative laid out by Schlingloff-Nemecz et al. (2023) is that children initially conceive of helping as a type of joint action. Here, multiple agents each direct their efforts towards bringing about a shared goal. Under such a conception, the agents’ utility functions are not hierarchically embedded, because the Helper does not altruistically attempt to increase the Helpee’s utility—instead, all agents derive a benefit from achieving the outcome. The individual goals of the agents involved are akin to subgoals in an action hierarchy, serving the purpose of bringing about the ultimate goal; the agents therefore do not hold fixed roles of benefit provider and beneficiary. In a joint action, agents should aim to minimize aggregate action costs and act jointly efficiently (cf. Török et al., 2019).

If children initially consider helping to be a type of joint action, in Block 1 they should have chosen to perform an action that would result in the least costly action sequence aggregated for all involved agents—i.e., move the obstacle that blocked the Helpee’s shorter path to a goal. The fact that they did not do so speaks against the joint action account.

Taken together, we conclude that preschool-aged children make use of a utility-based concept of helping. Specifically, our findings support an account according to which children take the goal of helping to be increasing a Helpee’s utility. The developmental trajectory of this understanding remains unclear: In Study 1, we found no evidence that a utility-based concept of helping is available to one-year-old infants, and in fact the results are consistent with the possibility that infants possess one of the simpler helping concepts described earlier (see Discussion in the Supplementary Material). Moreover, although some research has provided tentative evidence that adults leverage a second-order, utility-based concept to interpret the actions of agents in a highly parametrized environment (He et al., 2020; Ullman et al., 2009), not much is known about whether and under what conditions such concept is applied to make sense of everyday interactions. A complication arises from the fact that in pedagogical contexts, adults may not be so strict in applying criteria for identifying instances of helping: for example, they may praise a child for helping, even though the child’s action did not increase the Helpee’s utility (and perhaps even created more work for the caregiver). This mixed signaling may in turn lead to uncertainty in the child’s understanding. Further research is therefore needed to trace the acquisition of the concept of helping.

With the present studies, we aimed to gain insights into a question we believe has not received sufficient attention in the literature, despite the fact that research on the computations underlying children’s reasoning—especially in the social domain—has made great strides in recent years. Although a growing body of research suggests that young children and even infants have a sophisticated understanding of helping, not much is known about how exactly they represent such interactions. The present studies contribute to this research by indicating that while infants may not yet possess a mature understanding of helping, preschoolers, at least in some contexts, can apply a hierarchical naive utility calculus to interpret helping behaviors.

## ACKNOWLEDGMENTS

We thank Krisztina Andrási and Zsuzsanna Karap for their help with collecting data for Study 1.

## FUNDING INFORMATION

This work has received funding from the European Research Council under the European Union’s Horizon 2020 research and innovation program [grant agreement 742231 (PARTNERS), awarded to Gergely Csibra].

## Note

^1^ This is surprising, as the layout in the first test trial of Block 2 was almost identical to the familiarization layout, but was different in Block 1.

## Supplementary Material


