# Supplementary material for: Young Children’s Understanding of Helping as Increasing Another Agent’s Utility
Source: Open Mind (Camb). 2025 Jan 23;9:169–88. doi: 10.1162/opmi_a_00183 (PMC11793198; doi:10.1162/opmi_a_00183)
Supplement: Supplementary file 1 [file opmi-09-169-s001.docx]

**Young children's understanding of helping as increasing another agent’s utility**

**Supplementary Material**: **Details of Study 1**

## **Experiment 1A**

## **Methods**

#### ***Participants***

Twenty-four 12-month-old infants (13 male, age range: 11.2 – 13 m., mean age: 12.1 m) participated in Experiment 1A. An additional 17 infants were tested, but had to be excluded due to fussiness (n = 5), lack of attention to test events (n = 8), outside noise from a construction site during data collection (n = 3), and parental interference (n = 1). Participants in this and the following experiments were full-term infants with no reported health or developmental issues. They were recruited from the lab’s database and received a small toy for their participation. Before the experiment, caregivers were informed about the nature and possible consequences of the study, and gave informed consent for their child to participate. We obtained ethical approval for this and the following experiments from the university’s ethics committee.

#### ***Apparatus***

Infants were seated in their caregiver’s lap held by the hips in a darkened, soundproof room, 80 cm away from a 40-inch monitor. The stimuli were 2-D animated videos created using Adobe Animate CC software and presented with MATLAB (The MathWorks) using the Psychophysics toolbox extension (Brainard, 1997). Videos of the infants were recorded during the session. Infants’ looking behavior was coded on-line to determine when to start a new trial, and later manually coded off-line to measure looking time.

#### ***Procedure and stimuli***

Caregivers were instructed not to interact with the infants during the experiment. Their eyes were covered with opaque sunglasses. Before each trial, a short attention-getting clip was shown until the infant looked at the screen. Trials ended either when the infant looked away for a minimum of 2 seconds consecutively after the video had stopped, or if 8 seconds (familiarization) resp. 60 seconds (test) had elapsed since the end of the video.

*Familiarization.* Infants watched eight familiarization videos (Figure S.1, A). We presented two types of videos: “Solo” and “Helping”; the presentation order was SSHHSSHH. In half of the videos, the goal object was located on the top part of the screen, in the other half on the bottom; the order was counterbalanced.

In the videos, a goal object (strawberry) was always located on the far right side of the screen (either in the top or bottom corner). In the middle of the screen, there was a vertical barrier containing two openings. One of the openings – the one located closer to the goal – was blocked by a light blue door.

In the Solo familiarization videos (13 s), a character (the Helpee, a yellow circle with googly eyes directed rightward) appeared from the left side and moved toward the right (3 s), then paused in front of the barrier, in the middle between the two openings (1 s). Then, the Helpee moved through the unblocked opening towards the goal (5 s). After making contact with the strawberry, the Helpee bounced up and down (4 s).

The Helping familiarization videos (16 s) were similar, except that here, another character – the Helper (a green square with googly eyes directed leftward) – was located on the right side of the barrier, equidistant to the two openings and close to the strawberry. Again, the Helpee entered from the left and paused in front of the barrier (3 s). The Helper then moved toward the door, opened it, and returned to his initial position (5 s). The Helpee then approached the strawberry as in the Solo familiarization clips (8 s).

Key elements of the videos (the Helpee moving, beginning the goal approach through the opening in the barrier, reaching the goal; the door opening) were accompanied by sound effects.

*Test.* Infants received two test trials (Figure S.1, B): a Consistent and an Inconsistent trial (15 s each). The scenario in the test videos was similar to the one in the helping familiarization trials, except that now both doors were closed. The reward was located in the opposite location from the last familiarization trial (so, if it was on the bottom of the screen in the last familiarization video, it was at the top at test, and vice versa). In both videos, the Helpee again approached from the left (3 s) and paused in front of the barrier (1 s). Then, he briefly moved towards each of the doors (3 s). This was meant to highlight to infants that both possible paths were now blocked.

In the Consistent test event, the Helper opened the door that was closer to the goal (6 s). In the Inconsistent event, she opened the one further away from the reward. After this, in both events, the sound that, during familiarization, preceded the onset of the Helpee’s goal approach movement was played again, but now the video ended before the Helpee started moving.

We counterbalanced the location where the goal object was located in the first trial (top vs. bottom), the order of the Helpee’s partial approach movements in the test trials (to the upper opening first vs. to the lower opening first) and the order of test trials (Consistent first vs. Inconsistent first).


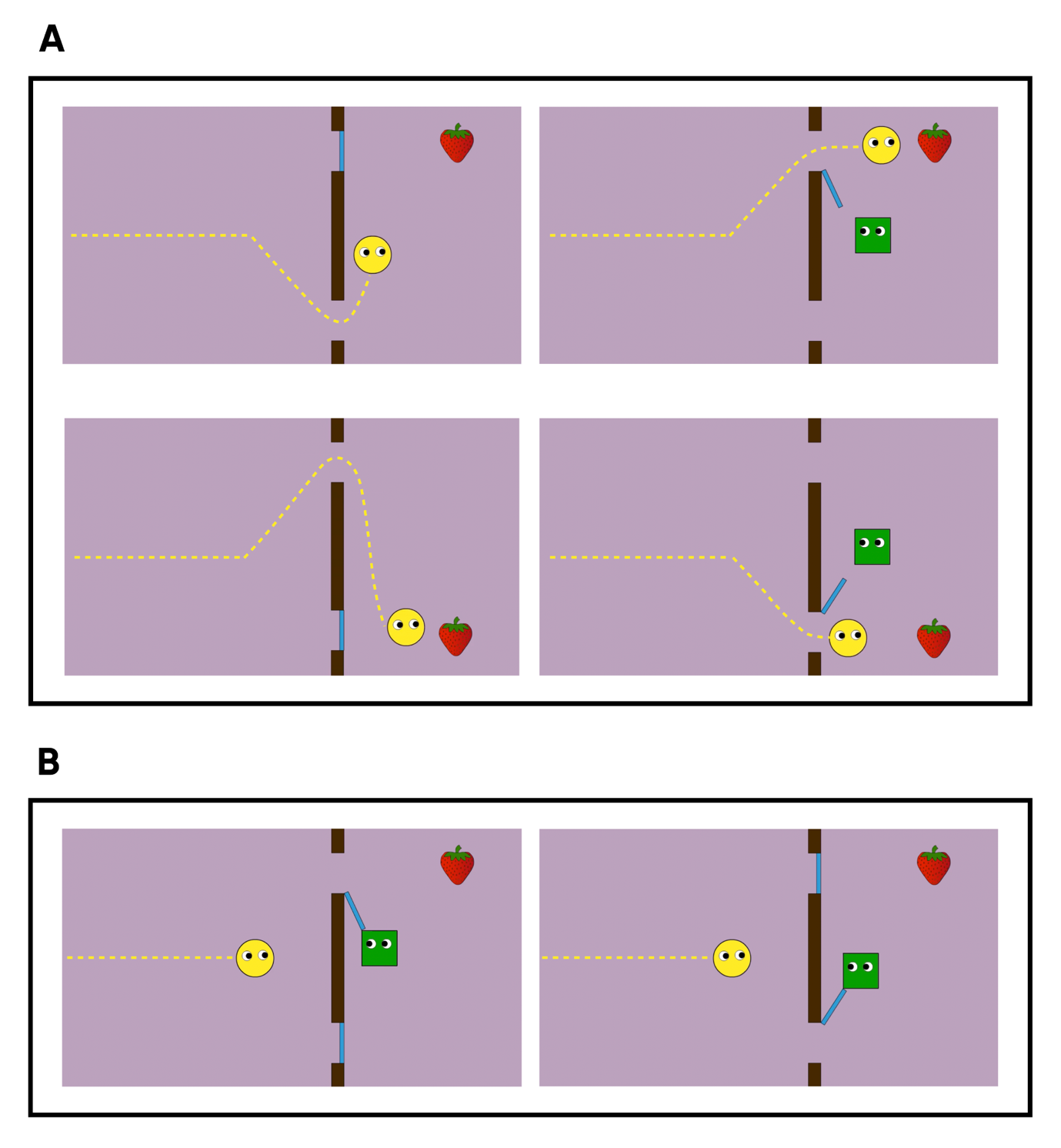

**Figure S.1.** Stimuli used in Experiment 1A. During Familiarization (A), the Helpee (yellow) approached the goal object. When alone, the Helpee had to take a longer path (left column); when the Helper (green) was present, he could take a shortcut. At Test (B), the Helper opened either the door that allowed the Helpee to take the short path (Consistent test event, left) or the long path (Inconsistent test event, right).

####

#### ***Coding and analyses***

Infants’ looking in the test trials was measured from the point of time when the two test events began to diverge, i.e. when the Helper started approaching one of the doors to open it. The looking behavior was manually coded off-line to measure looking times using the same criteria as online coding and reviewed for the pre-defined exclusion criteria (fussiness; parental interference; experimenter error; lack of attention during the test trials: i.e. failing to look for at least 50% of each trial in total, as well as at least 50% to each crucial door-opening action). The looking times of 50% of the participants was reanalyzed by an independent second coder who was blind to the hypothesis and to the condition of the stimuli shown. The recoded data were strongly correlated with the original data (*r* = 0.99, *p* < .001). Because of this high level of agreement, data from the first coder was used for analyses (in this and the following experiments).

The raw looking times were base-10 log-transformed for analyses (Csibra et al., 2016), but for descriptive statistics and plots we use the raw data. We conducted both Bayesian and frequentist statistical analyses. For the Bayesian analysis, we used the method recommended by Csibra et al. (2016) for looking-time data. For the frequentist statistical analyses, we conducted a paired sample two-tailed t-test on the data, and a 2x2 mixed ANOVA with Order as a between-subject and Trial as a within-subject factor to check for order effects. Statistical analyses and plotting were performed in R, version 4.3.1 (R Core Team, 2023).

### **Results**

There was no significant difference in looking times to the two test videos (*M*_consistent_ = 17.78 s, SD_consistent_ = 11.97 s; *M*_inconsistent_ = 20.75 s, *SD*_inconsistent_ = 13.38 s; *t*(23) = 1.24, *p* = 0.229), see Figure S.2. An ANOVA showed no significant main effects, but a significant effect of the Order by Trial interaction (*F*(1,22) = 7.61, *p* = 0.012). Subsequent t-tests showed that infants who saw the Consistent test trial first did not look significantly longer at either test event (*t*(11) = 0.92, *p* = 0.379), whereas those who saw the Inconsistent trial first looked significantly longer at the Inconsistent event (*t*(11) = 3.09, *p* = 0.01). This pattern suggests that there was an effect of Trial type, which interacted with infants’ tendency to look longer at the first test trial they saw.

In the Bayesian analysis, we obtained a BF of 0.54, which constitutes anecdotal evidence for the null hypothesis of no effect.


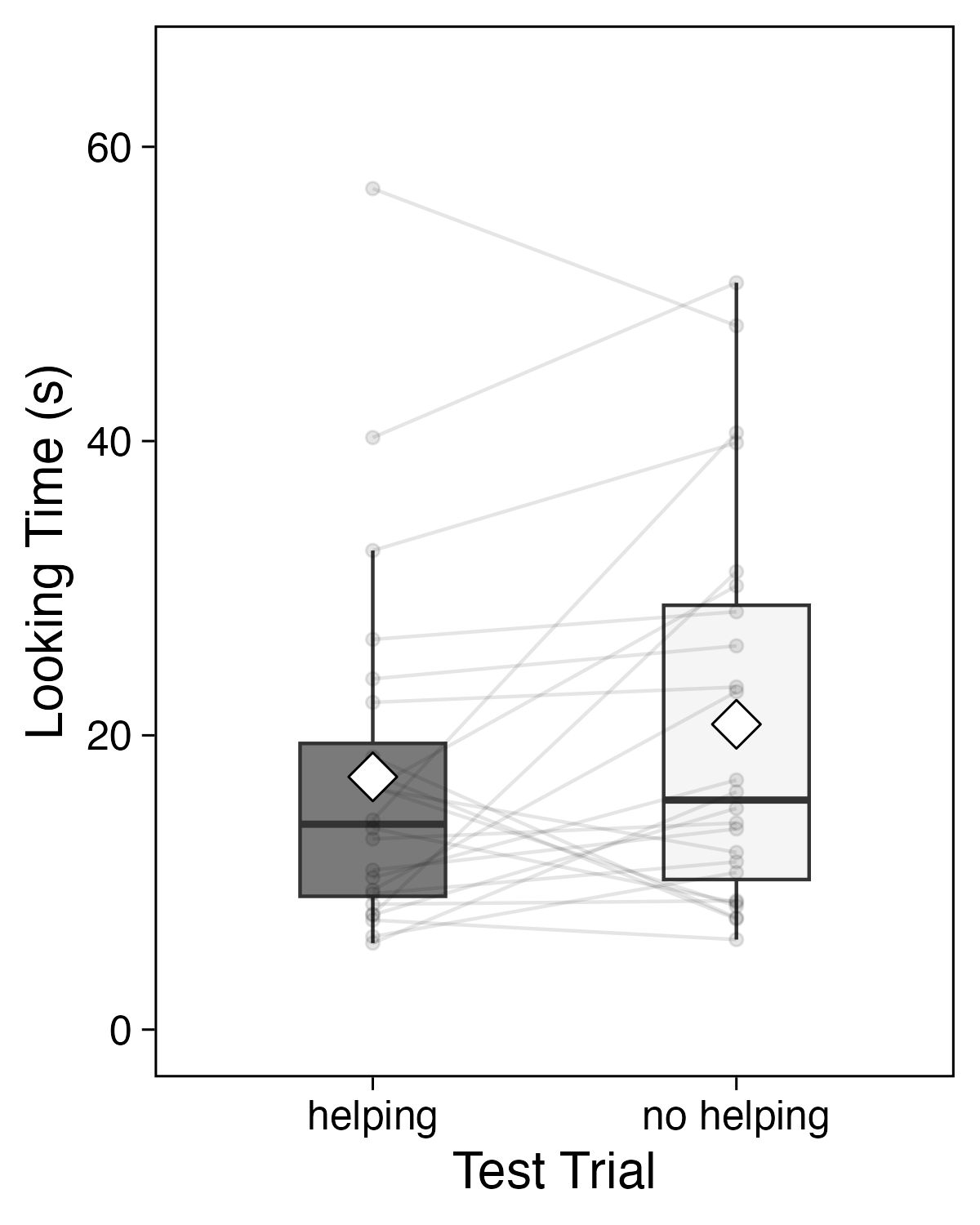


**Figure S.2.** Boxplot of average looking times (in seconds) toward the test events in Experiment 1A. Light grey lines connect the looking times of individual participants, white diamonds indicate means, horizontal lines indicate medians, boxes indicate middle quartiles, and whiskers indicate points within 1.5 times the interquartile range from the upper and lower edges of the middle quartiles.

###

### **Discussion**

In Experiment 1A, we aimed to test whether 12-month-old infants would expect an agent who previously reduced a Helpee’s action cost to select an action which allowed her to take the shortest path possible to her goal. The results we found were inconclusive. On the one hand, there was evidence for an order effect interacting with the effect of the test trial type, such that infants tended to look longer at the Inconsistent event, but only if this was presented in the first test trial. However, while this type of order effect is not uncommon in violation-of-expectation looking time designs (Baillargeon, 1987; Csibra et al., 1999; Liu et al., 2017; Mascaro & Csibra, 2012; Tatone et al., 2023), the looking difference between the test events in our experiment was not strong enough to yield a significant main effect. The Bayes factor similarly did not indicate a difference in looking times to the test trials, instead providing anecdotal evidence for the null hypothesis.

One way to interpret this pattern of results is that while some of the participants may have interpreted the stimuli as we intended and ascribed the goal of utility increase to an agent who had previously reduced a Helpee’s action cost, the task we posed to infants may have been too demanding. Specifically, infants had to compare two scenarios which both featured a utility increase for the Helpee, and had to compare the relative magnitude of this increase. Both actions at test, by allowing the Helpee to reach the object, could be considered well-formed instances of helping, in spite of the different costs the Helpee has to incur to reach his goal. If infants adopt this concept of helping, they should be able to appropriately identify helping when a scenario in which the Helpee does not receive any assistance is directly compared with one in which the Helper intervenes to reduce his costs. Experiment 1B was designed to examine this possibility.

## **Experiment 1B**

### **Methods**

#### ***Participants***

Twenty-four 12-month old infants (10 female, age range: 11.5 – 12.5 m., mean age: 12 m) participated in Experiment 1B. An additional 12 infants were tested but had to be excluded from the sample due to fussiness (n = 2), lack of attention to test events (n = 4), outside noise from a construction site during data collection (n = 2), experimenter error (n = 1), and having been retested a second time due to a scheduling error (n = 3). Recruitment, ethical approval, consent, and compensation were the same as in the previous experiment.

#### ***Apparatus***

The apparatus was the same as in Experiment 1A.

#### ***Procedure and stimuli***

The procedure was the same as in Experiment 1A.

*Familiarization.* Infants watched eight familiarization videos (Figure S.3, A). Again, two types of videos were shown: “Solo” and “Helping”; the presentation order was SH-SH-SH-SH. Within each of the Solo-Helping trial pairs, the physical layout of the scene was the same, but it varied across pairs. This allowed infants to observe the cost-reducing effect of the helping action directly, as the Helpee could take a relatively shorter path in the same environment after being helped, but also provided variability in the Helpee’s goal-approach motion paths across trials. The layout in the third and fourth pair of trials was the same as in the first and second, but rotated horizontally and vertically. The duration of trials varied, such that the first and third pair of videos were longer (due to a longer approach motion being necessary to reach the goal in this layout). There was always a goal object (strawberry) located somewhere in the scene, along with a wall containing two openings, of which one was obstructed by a black square block.


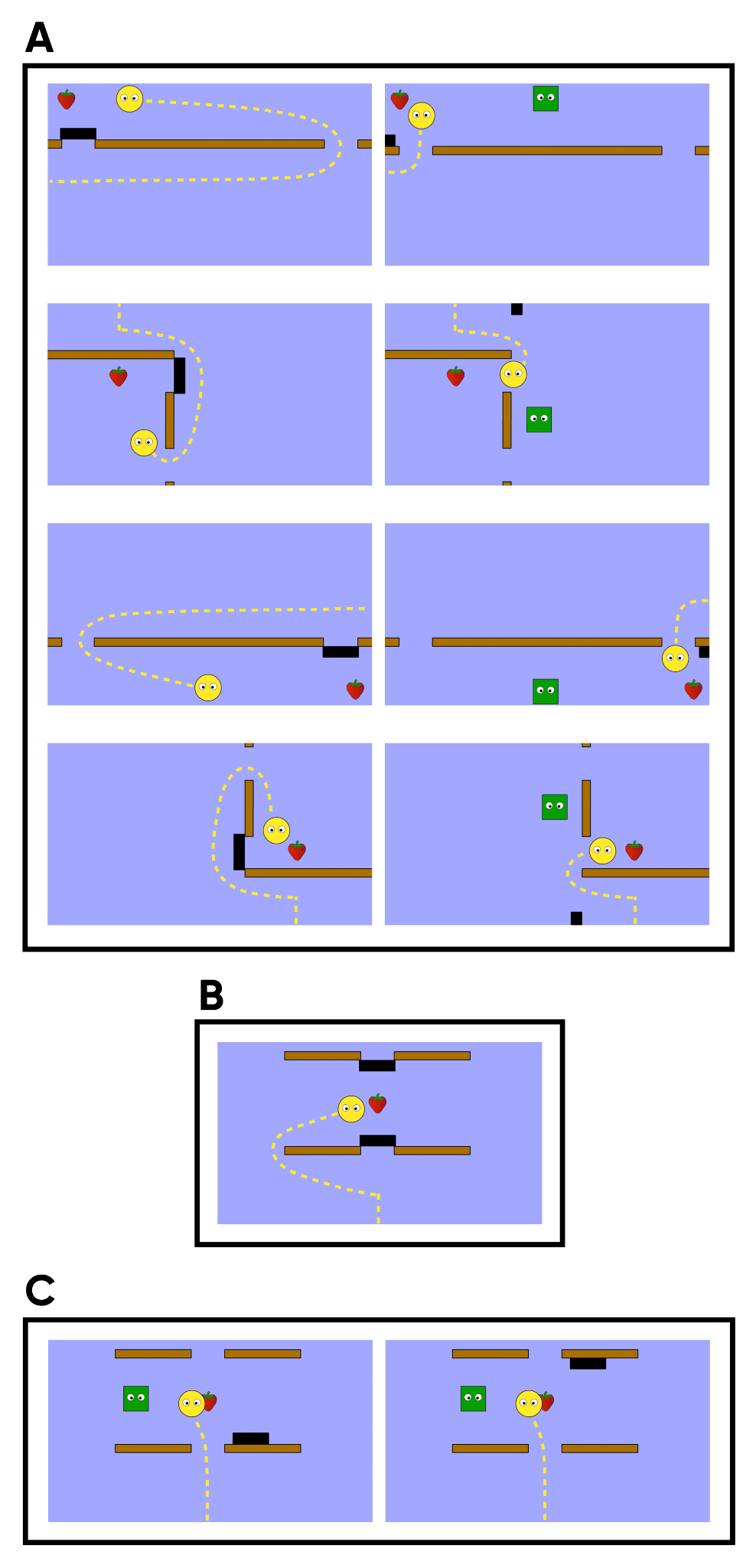


**Figure S.3.** Stimuli used in Experiment 1B. During Familiarization (A), the Helpee (yellow) approached the goal object. When alone, the Helpee had to take a longer path (left column); when the Helper (green) was present, he could take a shortcut. In the pre-test event (B), which had the same spatial layout as the test events, the Helpee approached the goal by detouring around the obstacle. At Test (C), the Helper either removed an obstacle that was in the way of the Helpee so that the Helpee could take the most direct path (Consistent test event, left), or removed an obstacle that was located elsewhere, even though the shortes path for the Helpee was already free (Inconsistent test event, right).

In the Solo familiarization videos, a character (the Helpee, a yellow circle with googly eyes as in Study 1) appeared on the screen (2 s) and paused (1 s). Then, the Helpee moved along the shortest possible trajectory through the non-obstructed opening (9/7 s) and finally reached the strawberry (1 s).

In the Helping familiarization videos, another character—the Helper (a green square with googly eyes)—was located in the middle between the openings in the barrier. Again, the Helpee entered and paused (2 s). The Helper then moved toward the opening that was obstructed by a block, pushed the block aside, and returned to his starting position (7/5 s), upon which the Helpee approached the strawberry as in familiarization (3/4 s).

*Pre-test.* The last trial of the familiarization phase served as a pre-test event. Here, the physical layout of the scene was the same as in the subsequent test events (save for the number of obstacles present), but the Helpee was alone, as in the Solo familiarization events (Figure S.3, B). There was a strawberry located at the center of the screen, and two horizontal barriers at equal distance from the strawberry, one above, one below it. Both barriers had openings that were blocked by an obstacle, respectively. The Helpee approached from the bottom of the screen and paused (3 s), then approached the strawberry by detouring around the lower barrier (4 s).

*Test.* After the familiarization and pre-test events, infants watched two test videos (Figure S.3, C): a Consistent and an Inconsistent trial (10 s each). Each test event featured both the Helpee and the Helper. The layout was the same as in the pre-test event, except now only one opening was blocked: In the Consistent trial, a block covered the gap in the lower barrier (thus obstructing the Helpee’s most direct path to the goal), while the upper barrier’s gap was unobstructed; conversely, in the Inconsistent trial, the opening in the upper barrier was covered by a block, while the gap in the lower barrier was free (such that the Helpee could approach the goal on a direct path). In both videos, the Helpee again approached from the bottom (2 s) and paused in front of the barrier. At this point, the Helper moved away the block from the opening (4 s), and the Helpee approached the strawberry on a straight, upward path (4 s). The behavior of the Helper was thus similar across the two test events, except that she moved upward or downward to remove the obstacle; the behavior (including the movement trajectory) of the Helpee was identical in the test events.

We counterbalanced the order of the familiarization videos (goal at the top vs. at the bottom of the screen in the first familiarization trial), and the order of test trials (Consistent first vs. Inconsistent first).

#### ***Coding and analyses***

The coding procedure, exclusion criteria, and data analyses were the same as in Experiment 1A.

Data recoded by an independent second coder (50% of participants) were strongly correlated with the original data (*r* = 0.98, *p* < .001).

### **Results**

There was no significant difference between the looking times to the test trials (*M*_consistent_ = 16.35 s, *SD*_consistent_ = 10.28 s; *M*_inconsistent_ = 15.92 s, *SD*_inconsistent_ = 8.84 s; *t*(23) = 0.38, *p* = .71), see Figure S.4. There were no significant main effects in the ANOVA, but the Order by Trial interaction was significant (*F*(1,22) = 7.53, *p* = .012). Subsequent t-tests showed that when splitting the sample by Order, looking time patterns did not differ significantly for either those infants who saw the Consistent trial first nor for those who saw the Inconsistent trial first (Consistent-first: *t*(11) = 1.89, *p* = .085; Inconsistent-first: *t*(11) = -2.006, *p* = .07). However, a paired t-test on looking times grouped by trial position (first vs. second trial presented to infants) showed that overall, infants looked significantly longer at the first event they saw (*M*_first_ = 18.45 s, *SD*_first_ = 10.87; *M*_second_ = 13.82, *SD*_second_ = 7.38; *t*(23) = 2.8, *p* = .01).

In the Bayesian analysis, we obtained a BF of 0.11, which constitutes substantial evidence for the null hypothesis.


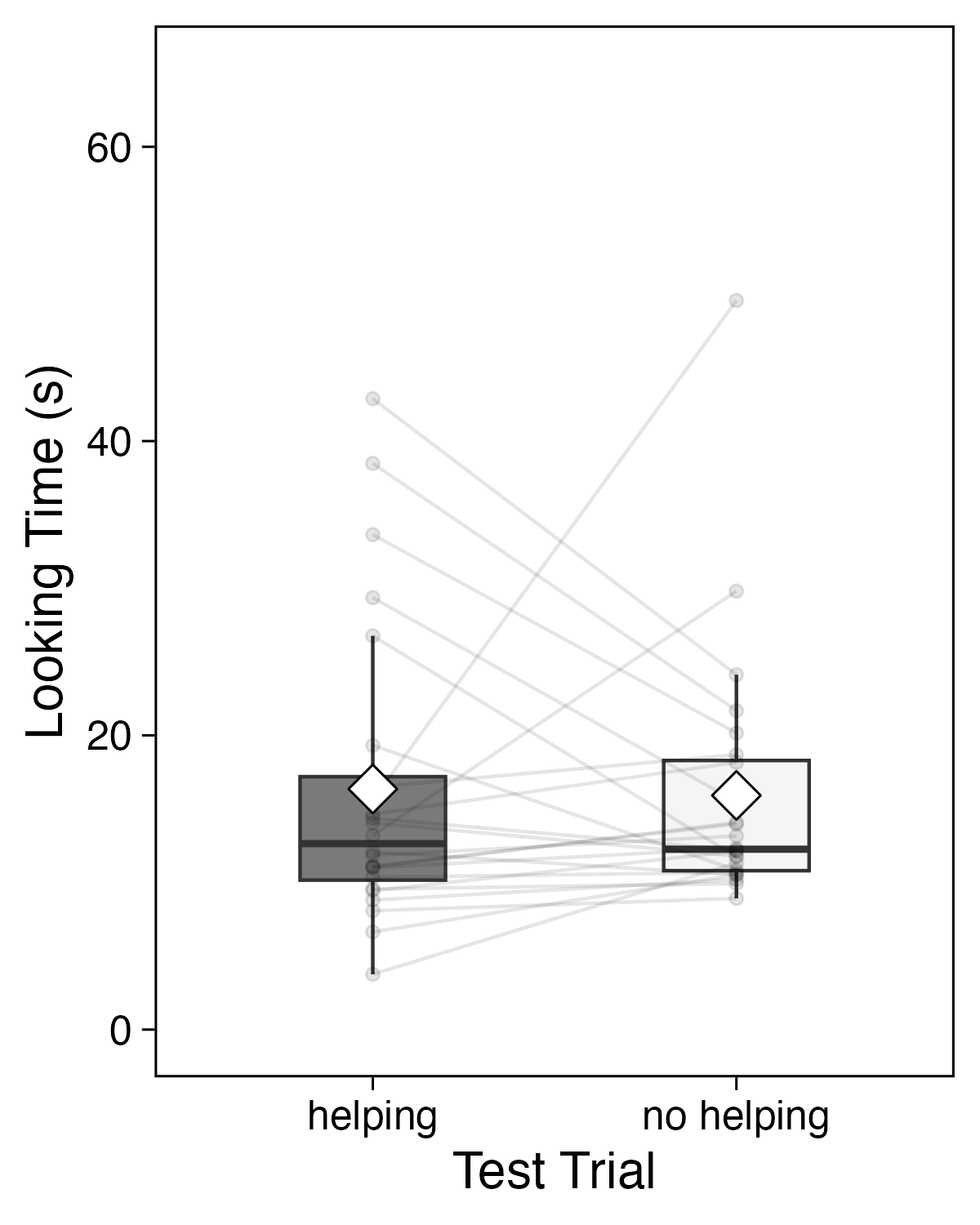


**Figure S.4.** Boxplot of average looking times (in seconds) toward the test events in Experiment 1B. Light grey lines connect the looking times of individual participants, white diamonds indicate means, horizontal lines indicate medians, boxes indicate middle quartiles, and whiskers indicate points within 1.5 times the interquartile range from the upper and lower edges of the middle quartiles.

###

### **Discussion**

The results of Experiment 1B did not support our hypothesis that infants take the goal of a Helper to be reducing the Helpee’s action cost. On the contrary, the Bayesian analysis provided evidence for the null hypothesis. Unlike in Experiment 1A, although we also found a significant interaction of Order and Trial type, this was due to an order effect: Infants simply looked longer at whichever test trial they saw first.

We reasoned that the task posed to participants in Experiment 1B would be easier than the one in Experiment 1A, as infants would have only to discriminate which of two actions reduced the costs of the Helpee (by whichever amount). If this was the case, they should have looked longer to the Inconsistent test trial, where the Helper performed an action that, despite looking similar to the helping action, did not have an effect on the Helpee’s goal-directed action However, we did not find the predicted response pattern.

One possibility for this result is that the stimuli were overall too challenging for infants, such that they failed to establish goal representations for any of the agents. We showed infants a helping interaction in four different spatial layouts (see Figure S.3), which we hoped would help infants represent the figures as efficient and goal-directed agents (Csibra, 2008; Csibra et al., 1999). It may be, however, that infants’ working memory capacities were overly burdened by processing the changing environments and figures’ movements within them. To rule out the possibility that participants were simply confused and did not reason about agents’ goals and efficiency at all, we ran Experiment 1C as a control condition.

##

## **Experiment 1C**

### **Methods**

#### ***Participants***

Twenty-four 12-month-old infants (13 male, age range: 11.5 – 12.5 m, mean age: 11.9 m) participated in Experiment 1C. An additional 16 infants were tested but had to be excluded due to fussiness (n = 4), failure to meet the pre-defined attentiveness criteria (n = 5) experimenter error (n = 4), parental interference (n = 2) and technical problems (n = 1). Recruitment, ethical approval, consent, and compensation were the same as in the previous experiments.

#### ***Apparatus***

The apparatus was the same as in Experiments 1A and 1B.

#### ***Procedure and stimuli***

The procedure was the same as in Experiments 1A and 1B.

The stimuli were the same as in Experiment 1B, save for the test trials (Figure S.5, C). In both test trial videos (11 s each), the block was in front of the opening in the lower barrier, thus obstructing the Helpee’s direct path to the reward, and the Helper pushed this block aside (as in the Consistent test trial in Experiment 1B). In the Consistent trial video, the Helpee approached her goal in the most direct path, moving straight upward (4 s). In the Inconsistent trial video, the Helpee approached the goal by detouring around the side of the barrier, moving along the same curvilinear path as in the pre-test trial where the direct path had been blocked (4 s). To equate the duration of the two videos, the Helpee moved faster in the Inconsistent trial, as here her path was longer.

#### ***Coding and analyses***

The coding procedure, exclusion criteria, and data analyses were the same as in Experiments 1A and 1B. The recoded data were again strongly correlated with the original data (*r* = 0.99, *p* < .001).


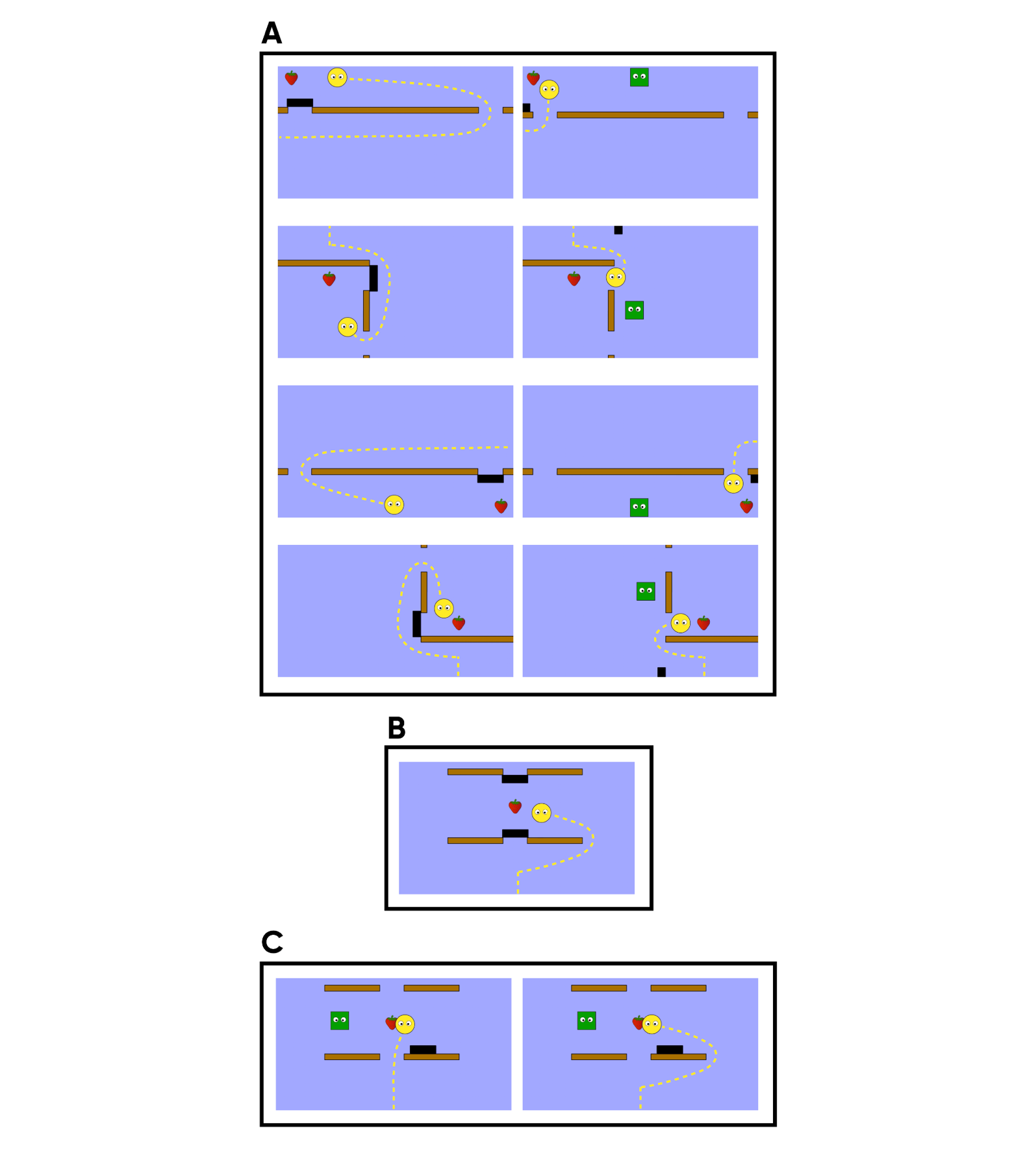


**Figure S.5.** Stimuli used in Experiment 1C. The Familiarization (A) and pre-test event (B) were the same as in Experiment 1B. At Test (C), the Helper helped in both test trials, while the Helpee approached his goal either efficiently, on the most direct path (Consistent test event, left), or inefficiently, by detouring around the side of the wall (Inconsistent test event, right).

### **Results**

Infants looked longer to the Inconsistent compared to the Consistent test event (*M*_inconsistent_ = 17.32 s, *SD*_inconsistent_ = 16.54 s, *M*_consistent_ = 10.02 s, *SD*_consistent_ = 7.59 s, *t*(23) = 2.6, *p* = .016), see Figure S.6. A 2x2 mixed ANOVA with Order as between-subject and Trial as within-subject factor showed only a significant main effect of Trial (*F*(1,22) = 6.5, *p* = .018), there was no significant effect of Order (*F*(1,22) = 1.81, *p* = .193) and no significant Order by Trial interaction (*F*(1,22) = 0.07, *p* = .801).

In the analysis using Bayesian statistics, we obtained a BF of 323.59, which constitutes strong evidence in favor of the hypothesis.


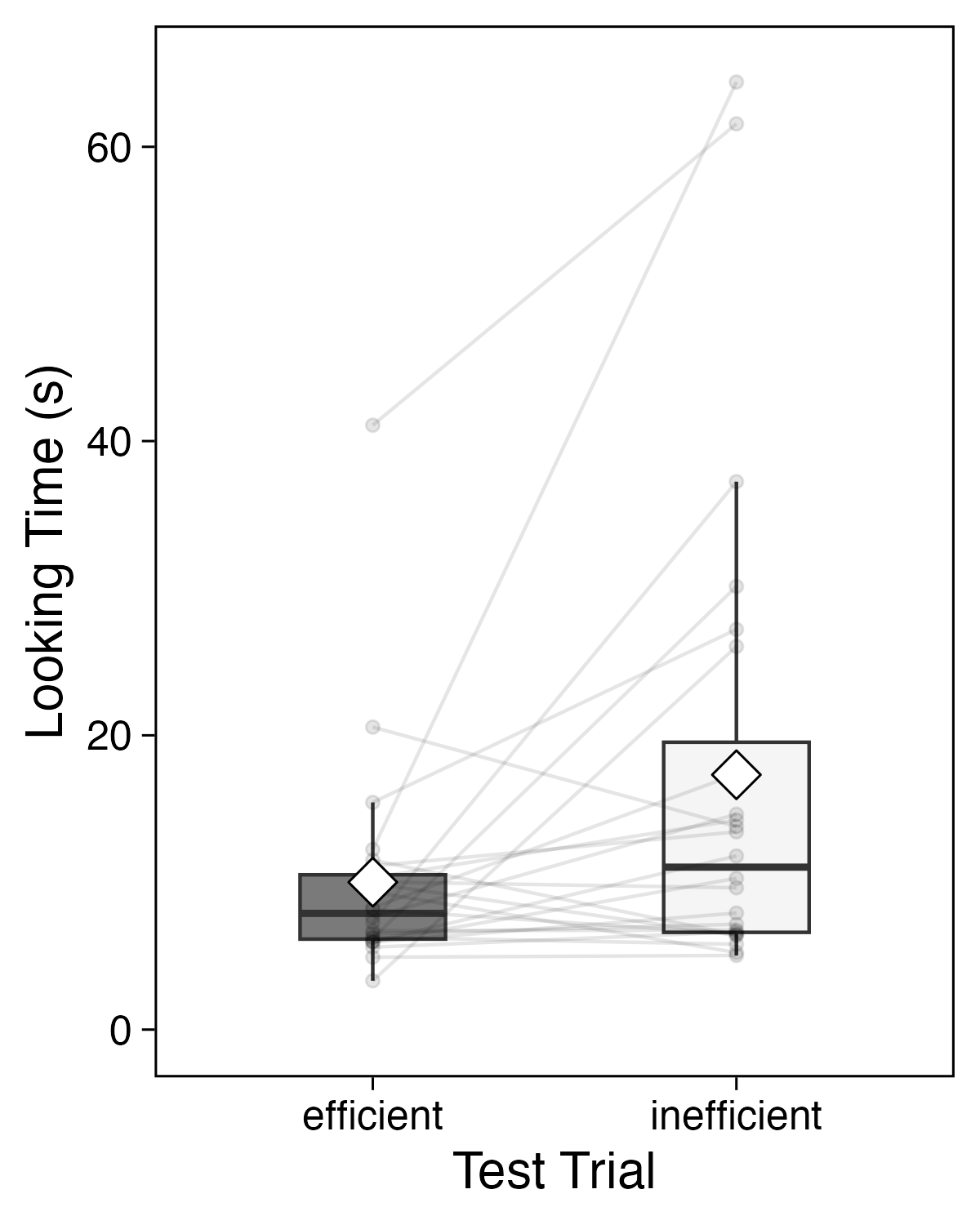


**Figure S.6.** Boxplot of average looking times (in seconds) toward the test events in Experiment 1C. Light grey lines connect the looking times of individual participants, white diamonds indicate means, horizontal lines indicate medians, boxes indicate middle quartiles, and whiskers indicate points within 1.5 times the interquartile range from the upper and lower edges of the middle quartiles.

### **Discussion**

As predicted, 12-month-old infants looked longer when an agent, the Helpee, moved towards the goal object in an inefficient manner after having previously done so efficiently, showing that infants succeeded in attributing the goal of approaching the strawberry to the Helpee.

This result replicates previous findings that preverbal infants expect agents to adhere to the principle of efficiency. It also extends these previous findings by demonstrating that one-year-olds can also set up a goal representation in a context where multiple agents perform distinct actions. The finding speaks against the possibility that infants in Experiment 1B did not ascribe a helping goal to the Helper because the stimuli were generally too complex for them to track.

## **General Discussion of Study 1**

The aim of the experiments reported here was to investigate whether infants understand helping as an action whose goal is to lower or minimize another agent’s action costs. The results, taken together, do not support this hypothesis. Infants’ looking behavior in Experiment 1A tentatively suggested that they may have found it less consistent with a Helper’s previous behavior when he did not minimize the Helpee’s action cost, but that this effect was masked by an additional order effect. However, Experiment 1B provided evidence for the null hypothesis: Infants did not distinguish between an event where the Helper performed a utility-increasing action and a similar-looking non-helpful action. Finally, Experiment 1C demonstrated that infants familiarized to the same stimuli as those in Experiment 1B successfully ascribed a non-social, instrumental goal to the Helpee in this scenario, ruling out the possibility that the stimuli or experimental procedure failed to elicit any kind of goal attribution.

There are different explanations for these results. One option is that infants possess a mature understanding of helping, but the stimuli or experimental design that we used were not well-suited to prompt participants to apply it. For instance, the cost differences the Helpee would incur as a result of being helped may have been too small to be salient for infants.

Another possibility is that infants at the age we tested struggled with the means-ends-reasoning required by our task, i.e., understanding that moving an obstacle freed a relatively shorter path for another agent (see e.g. Sommerville & Woodward, 2005; Woo & Spelke, 2023). They may therefore have attributed only first-order goals to both agents (Helpee: reach goal object, Helper: move a door/block), towards which the agents behaved efficiently (save for the Helpee in the Inconsistent trial of Experiment 1C). Ascribing a hierarchical means-ends structure, where subgoals are merely performed in the service of facilitating an ultimate goal, may be especially difficult in a social context, as infants have to override a potential prior assumption that agents tend to perform actions to acquire personal benefits. However, even for non-social action contexts, it has not been directly tested whether infants understand that means or subgoals can serve the sole purpose of making the overall action sequence less costly.

A further possibility is that at 12 months of age, infants cannot understand helping at all. However, this conclusion would be at odds with the large body of literature suggesting that even much younger infants prefer helpful agents (but see Lucca et al., 2024), and that this preference depends on the intentions infants ascribe to them (Woo et al., 2023).

Finally, another option is that initially, infants conceive of helping in a different way, as laid out in Schlingloff-Nemecz et al. (2023) and the discussion section of the present manuscript. If, for instance, young infants possess a concept of helping as enabling or as joint action, they could not have succeeded in our experiments. With a concept of helping as enabling, an observer would only consider an action helping if it allowed the Helpee to reach a previously inaccessible goal. In our stimuli, the Helper did not perform an enabling action in any of the familiarization events; in the test events of Experiment 1A, both of the Helper’s actions were enabling; and at test in Experiment 1B, neither was. Therefore, a possessor of an enabling concept would not have set up a representation of helping during familiarization, and would not have distinguished between test events. With a concept of helping as joint action, on the other hand, an observer might rely on the presence of particular social interaction cues, which help establish an interpretive prior that the agents in the scene are participating in a collaborative endeavor. Our stimuli were largely void of such cues: The agents did not engage in communication or eye contact, were not in close physical proximity to one another while pursuing their respective goals, and did not act on the same objects. A possessor of a joint action concept may thus not have related the behaviors of Helper and Helpee, and accordingly not have established a shared goal for them.

In conclusion, our results don’t support the hypothesis that infants have, or recruit, a mature concept of helping when observing a helping event. It is therefore still an open question how infants understand helping actions, and how this understanding emerges in ontogeny. Follow-up research will have to uncover when, and how, young children come to acquire an understanding of helping as increasing the Helpee’s utility, what its predecessor—if infants initially rely on a simpler concept of helping—looks like, and how the former develops from the latter.

**References**

Baillargeon, R. (1987). Object permanence in 3½- and 4½-month-old infants. *Developmental Psychology*, *23*(5), 655–664. https://doi.org/10.1037/0012-1649.23.5.655

Brainard, D. H. (1997). The Psychophysics Toolbox. *Spatial Vision*, *10*, 433–436.

Csibra, G. (2008b). Goal attribution to inanimate agents by 6.5-month-old infants. *Cognition*, *107*(2), 705–717. https://doi.org/10.1016/j.cognition.2007.08.001

Csibra, G., Gergely, G., Bı́ró, S., Koós, O., & Brockbank, M. (1999). Goal attribution without agency cues: The perception of ‘pure reason’ in infancy. *Cognition*, *72*(3), 237–267. https://doi.org/10.1016/S0010-0277(99)00039-6

Csibra, G., Hernik, M., Mascaro, O., Tatone, D. & Lengyel, M. (2016). Statistical Treatment of Looking-Time Data. Developmental Psychology, 52(4), 521-536. https://doi.org/10.1037/dev0000083

Liu, S., Ullman, T. D., Tenenbaum, J. B., & Spelke, E. S. (2017). Ten-month-old infants infer the value of goals from the costs of actions. *Science*, *358*(6366), 1038–1041. https://doi.org/10.1126/science.aag2132

Lucca, K., … Hamlin, K. (2024). *Infants’ Social Evaluation of Helpers and Hinderers: A Large-Scale, Multi-Lab, Coordinated Replication Study*. https://doi.org/10.31234/osf.io/qhxkm

Mascaro, O., & Csibra, G. (2012). Representation of stable social dominance relations by human infants. *Proceedings of the National Academy of Sciences*, *109*(18), 6862–6867. https://doi.org/10.1073/pnas.1113194109

Schlingloff-Nemecz, L., Tatone, D. & Csibra, G. (2023). The Representation of Third-Party Helping Interactions in Infancy. *Annual Review of Developmental Psychology 5*, 67-88. https://doi.org/10.1146/annurev-devpsych-120321-033548

Sommerville, J. A., & Woodward, A. L. (2005). Pulling out the intentional structure of action: the relation between action processing and action production in infancy. *Cognition*, *95*, 1- 30. doi:10.1016/j.cognition.2003.12.004

Tatone, D., Schlingloff-Nemecz, L., & Pomiechowska, B. (2023). Infants do not use payoff information to infer individual goals in joint-action events. *Cognitive Development*, *66*, 101329. https://doi.org/10.1016/j.cogdev.2023.101329

Woo, B. M., & Spelke, E. S. (2023). Infants and toddlers leverage their understanding of action goals to evaluate agents who help others. *Child Development*, *94*(3), 734-751.

Woo, B. M., Tan, E., Yuen, F. L., & Hamlin, J. K. (2023). Socially evaluative contexts facilitate mentalizing. *Trends in Cognitive Sciences*, *27*(1), 17–29. https://doi.org/10.1016/j.tics.2022.10.003
